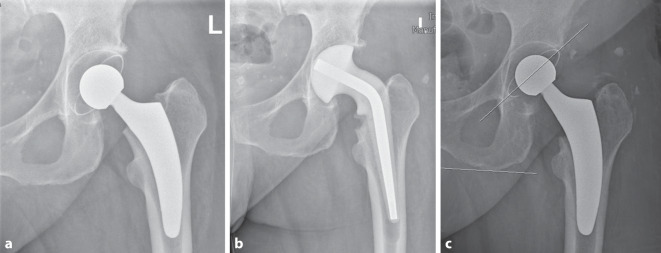# Herausforderung: Revision eines fest eingewachsenen kurvierten Kurzschaftes in der Hüftendoprothetik: Vorstellung eines neuen, kurvierten Extraktions-Meißel-Systems

**DOI:** 10.1007/s00064-022-00775-6

**Published:** 2022-05-29

**Authors:** Karl Philipp Kutzner, Karl Stoffel, Josef Hochreiter

**Affiliations:** 1 Gelenkzentrum Rhein-Main, Wilhelmstr. 30, 65183 Wiesbaden, Deutschland; 2 Klinik für Orthopädie und Unfallchirurgie, St. Josefs Hospital Wiesbaden, Wiesbaden, Deutschland; 3 Zentrum für Orthopädie und Unfallchirurgie, Universitätsmedizin Mainz, Mainz, Deutschland; 4 Orthopädie Klinik, Hüfte/Beckenchirurgie, Universitätsspital Basel, Basel, Schweiz; 5 Orthopädie, Ordensklinikum Linz, Linz, Österreich

**Keywords:** Revision, Hüft-TEP, Ausbau, Infektion, Instrumentarium, Revision surgery, THA, Infection, Removal, Instruments

## Abstract

**Operationsziel:**

Sichere und knochensparende Extraktion eines fest eingewachsenen kurvierten Kurzschaftes ohne die Notwendigkeit eines transfemoralen Zuganges.

**Indikationen:**

Revision eines fest eingewachsenen Kurzschaftes beispielsweise bei periprothetischer Infektion oder Malposition.

**Kontraindikationen:**

Korrekte Platzierung der Konusschablone nicht möglich.

**Operationstechnik:**

Wahl eines Standardzugangs zum Hüftgelenk. Luxation des Gelenkes. Abschlagen des einliegenden Kopfimplantats. Sorgfältiges Freipräparieren des Konus und der lateralen Schulter. Aufbringen und Befestigen der Konusschablone. Beginn des vorsichtigen Einschlagens der Eröffnungsmeißel, zunächst lateral, anschließend ventral und dorsal über die Führungen der Schablone. Es ist darauf zu achten, dass stets der Schliff vom Implantat weg zeigt. Anschließend Wiederholung des Vorganges mit den Startermeißeln in gleicher Reihenfolge. Entfernung der Schablone. Vorsichtiges Einschlagen der Finalen Meißel in gleicher Reihenfolge. Versuch einer Schaftextraktion mittels Ausschlaginstrumentarium. Fakultativ Wiederholung des Gesamtvorganges. Zur Vermeidung von Frakturen, erst nach Mobilisation des Interfaces lateral, ventral und dorsal, Freilegung des Interfaces auch medial mittels medialer Meißel in der entsprechenden Reihenfolge. Abschließend Schaftextraktion.

**Weiterbehandlung:**

Mobilisierung und Belastungsaufbau gemäß der gewählten Revisionsimplantate und Verankerungstechnik.

**Ergebnisse:**

Das beschriebene Vorgehen hat sich in der klinischen Praxis in den 3 Autorenkliniken in insgesamt 14 Fällen bewährt. In 3 (21,4 %) Fällen musste trotz Nutzung des Extraktions-Meißel-Systems zusätzlich ein transfemoraler Zugang bzw. eine Fensterung erfolgen, um das Schaftimplantat zu entfernen. Als Revisionsimplantate wurden in über der Hälfte der Fälle (57,8 %) primäre Geradschäfte verwendet, in 4 Fällen (36,4 %) konnte erneut ein zementfreier Kurzschaft verwendet werden.

## Abstract (en)

**Objective:**

Safe and bone-conserving extraction of a well-fixed curved short stem without the necessity of a transfemoral approach.

**Indications:**

The revision of a well-fixed curved short stem, for example, due to periprosthetic infection or malposition. Meticulous preparation of the cone and the lateral shoulder of the stem.

**Contraindications:**

Correct placement of the chuck not possible.

**Surgical technique:**

Choice of a standard approach to the hip joint. Luxation. Removal of the implanted head. Preparation of the proximal femur and removal of bone at the stem shoulder. Attachment of the chuck to the cone. Insertion of the “prestarter” chisels through the guided slots of the chuck, starting with the lateral chisel, followed by the ventral and dorsal chisel. The cut must point outwards away from the implant. Repetition of this procedure using the “starter” chisels in the same order. Removal of the chuck. Careful insertion of the “final” chisels in the same order. Trial of a stem extraction using an extraction tool. Optional repetition of the whole procedure. In order to avoid fractures, opening of the medial interface only after preparation laterally, ventrally and dorsally, by careful insertion of the medial chisels in the respective order alongside the calcar. Finally, extraction of the stem.

**Postoperative management:**

Postoperative protocol according to the respective revision implants and fixation technique used.

**Results:**

The described procedure has proven successful in clinical practice in the three author affiliations in a total of 14 cases. In 3 (21.4%) cases, despite the use of the extraction chisel system, an additional transfemoral approach or fenestration had to be performed to remove the short stem. Primary straight stems were used in over half of the cases (57.8%) as revision implants, whereas in 4 cases (36.4%) a cementless short stem could again be used.

## Vorbemerkungen

Kurzschäfte haben in der primären Hüftendoprothetik in den vergangenen Jahren insbesondere in Europa stetig an Popularität gewonnen. Bereits heute liegt der Anteil der in Deutschland implantierten Kurzschäfte bei über 10 % aller primären Hüft-Totalendoprothesen(TEP)-Versorgungen [1].

Mit der zunehmenden Zahl an implantierten Kurzschäften, häufen sich jedoch auch Komplikationen mit dieser Art von Implantaten, und auch die Revisionsoperationen von Kurzschäften geraten zunehmend in den Fokus [2].

Zu den häufigsten Komplikationen gehören die aseptische Lockerung und der periprothetische Infekt.

Aufgrund der Unterschiede im Design der Kurzschäfte im Vergleich zu den meisten konventionellen Geradschäften stellen Revisionsoperationen von fest eingewachsenen Kurzschäften eine besondere Herausforderung dar.

Moderne Kurzschäfte weisen zumeist ein kurviertes Design auf, sind konisch und werden kalkar-geführt, also „round the corner“ implantiert [3]. Es handelt sich um zementfreie, beschichtete Implantate, welche nach initialer Primärstabilität durch „Press-fit“-Einschlagen in das proximale Femur sekundär in den ersten 6 bis 10 Wochen in den Knochen einwachsen und damit die Sekundärstabilität erlangen [4].

Im Falle eines periprothetischen Infektes (Spätinfekt) müssen zumeist alle Implantate vollständig entfernt werden. Ein fest in den Knochen eingewachsener Schaft stellt, bedingt durch das kurvierte Schaftdesign, in der Kurzschaftendoprothetik eine große Herausforderung dar.

Herkömmliche, gerade Meißelsysteme können hierbei aufgrund einer erhöhten Perforationsgefahr nur eingeschränkt zur Anwendung kommen. Eine endofemorale Explantation mittels der vorhandenen geraden Meißelsysteme ist häufig nicht möglich. Nicht selten muss daher ein aufwendiger transfemoraler Zugang erfolgen mit zum Teil erheblichen negativen Folgen für den Knochenerhalt, die postoperative Stabilität und die Nachbehandlung der Patienten [5].

Nachfolgend wird daher ein neues kurviertes Extraktions-Meißel-System inklusive Konusschablonen mit Führungsschlitzen speziell für die Kurzschaftendoprothetik vorgestellt. Hierbei handelt es sich um ein System, welches an das Design des optimys-Kurzschaftes (Fa. Mathys, Bettlach, Schweiz) angepasst ist.

## Operationsprinzip und -ziel

Geführtes Einbringen von speziell kurvierten Einmalmeißeln aus geschliffenem, rostfreiem Stahl über Führungsschlitze einer Konusschablone in systematischer Reihenfolge. Hierbei werden zunächst schmale Eröffnungsmeißel („Prestarter“ und „Starter“) genutzt. Nach Entfernen des Konusadapters kann dann mittels der „Finalen“ Meißel das Schaftimplantat aus dem Knochenverbund gelöst werden. Das Ziel ist die sichere und knochensparende Entfernung eines fest eingewachsenen Schaftes ohne die Notwendigkeit aufwendiger transfemoraler Zugänge bzw. Knochenfensterungen.

## Vorteile

- Sicheres Einbringen der Extraktionsmeißel über Führungsschlitze der Schablone
- Formstabile Meißel mit unterschiedlichen Radien, angepasst an das Schaftdesign
- Drei verschiedene Fixierungspositionen der Meißel in der Länge auf dem Handgriff ermöglichen eine hohe Flexibilität mit maximaler Kraftübertragung
- Minimaler Knochenverlust durch präzise Trennung von Knochen und Implantat auf allen Seiten
- Zum Teil deutliche Zeitersparnis

## Nachteile

- Erhöhte Kosten durch zusätzliches Instrumentarium und Material (insgesamt maximal 10 Einmalmeißel)

## Indikationen

- Notwendigkeit einer Revision eines fest eingewachsenen kurvierten Kurzschaftes beispielsweise bei periprothetischer Infektion oder Malposition

## Kontraindikationen

- Korrekte Platzierung der Schablone auf dem Implantatkonus nicht möglich
- Nicht-kurviertes Kurzschaftdesign

## Patientenaufklärung

- Aufklärung hinsichtlich Hüft-TEP-Revision mit Explantation der Implantate
- Gegebenenfalls zusätzlich transfemoraler Zugang bei frustranem Versuch der Prothesenentfernung

## Operationsvorbereitungen

- Hautdesinfektion, sterile Abdeckung der Hüfte und des Beines
- Platzierung des Bildverstärkers auf der ipsilateralen Seite
- Präoperative Planung hinsichtlich des Wiedereinbaus von Implantaten

## Instrumentarium

**Figure 1:**
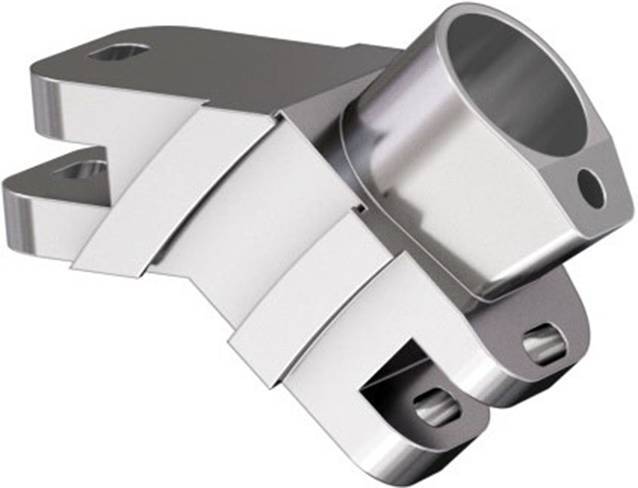

**Figure 2:**
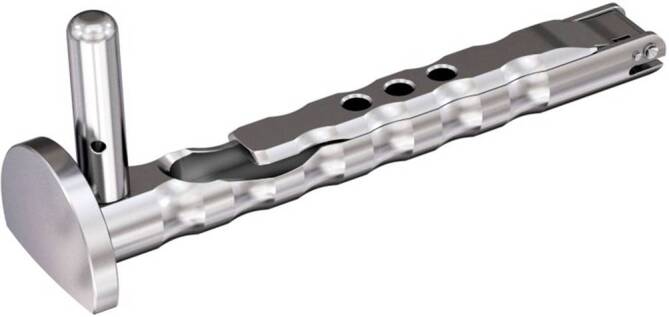

**Figure 3:**
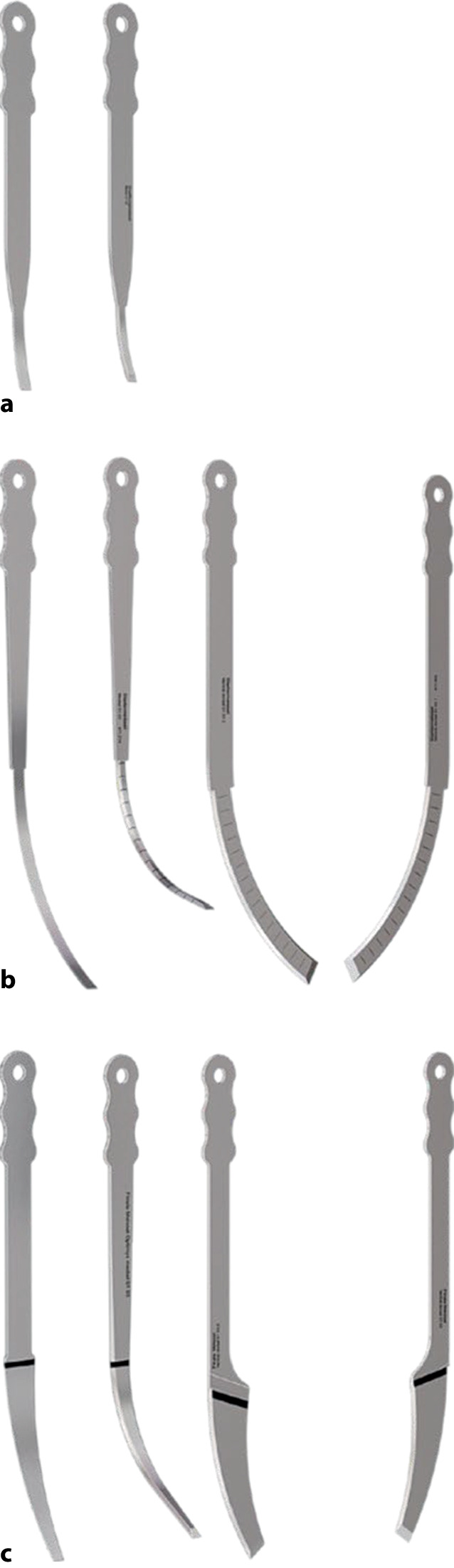

- Standardinstrumentarium für die Hüft-TEP-Revision
- Konusschablone (Fa. Mathys, Robert-Mathys-Str. 5, CH-2544 Bettlach, Schweiz) (Abb. 1) inklusive Fixationsschraube und Schraubendreher. Eine unterschiedliche Schablonengröße für jede der verschiedenen Schaftgrößen und Offsetversionen ist notwendig (insgesamt 24 verschiedene Größen)
- Handgriff mit 3 verschiedenen Positionsoptionen für die jeweiligen Meißel (Abb. 2) (Fa. Gomina, Raiftstr. 4, CH-3989 Niederwald, Schweiz)
- Eröffnungsmeißel („Prestarter“) (lateral, medial) (Abb. 3a) (Fa. Gomina)
- Startermeißel (lateral, ventral, dorsal, medial) (Abb. 3b) (Fa. Gomina)
- Finale Meißel (lateral, ventral, dorsal, medial) (Abb. 3c) (Fa. Gomina)

## Anästhesie und Lagerung

- Allgemeinanästhesie oder Spinalanästhesie
- Rücken- und Seitenlage möglich
- Adäquates Auslagern nach Luxation muss möglich sein
- Intraoperative Durchleuchtung des Hüftgelenkes sollte möglich sein

## Operationstechnik

**(**Abb. 4, 5, 6, 7, 8, 9, 10, 11, 12, 13, 14, 15 und 16**)**

**Figure 4:**
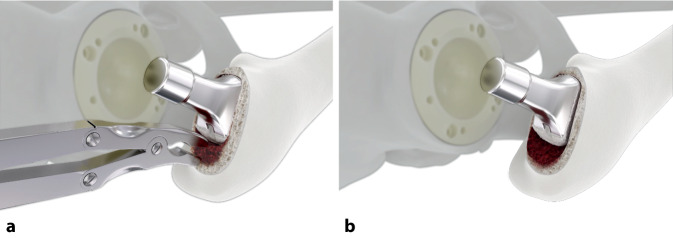

**Figure 5:**
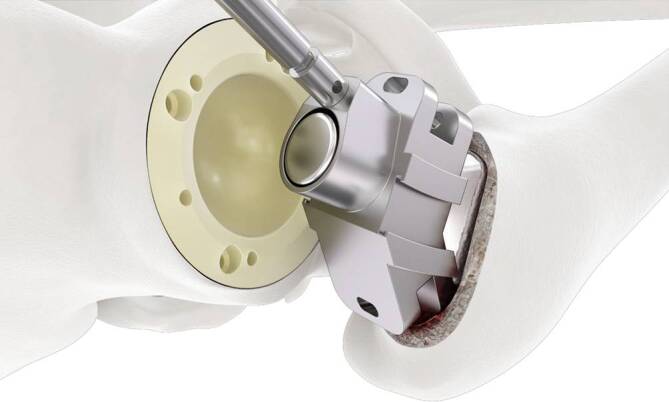

**Figure 6:**
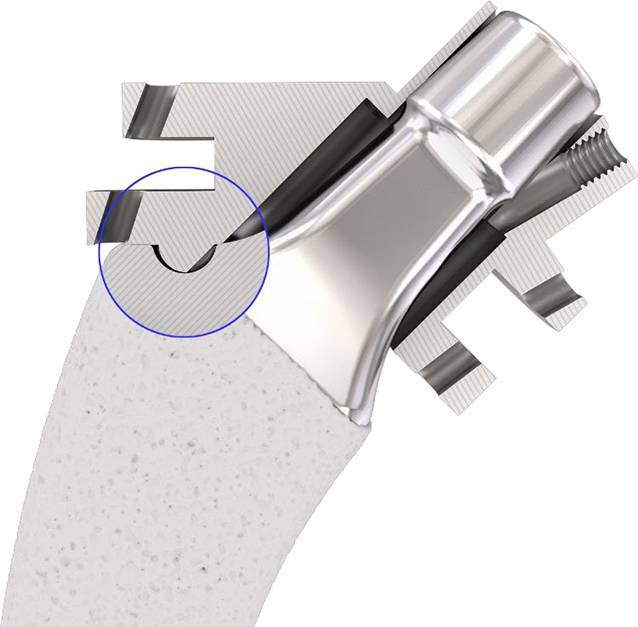

**Figure 7:**
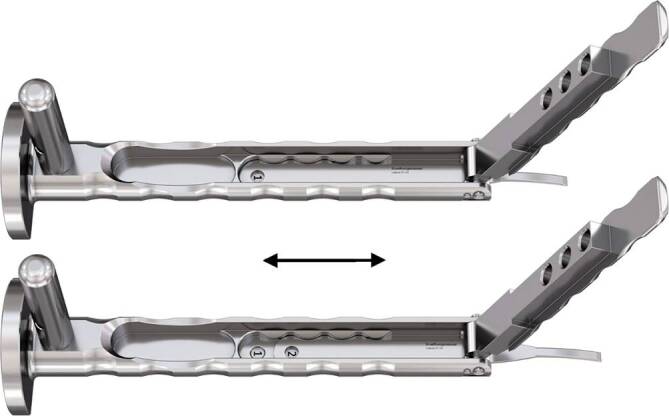

**Figure 8:**
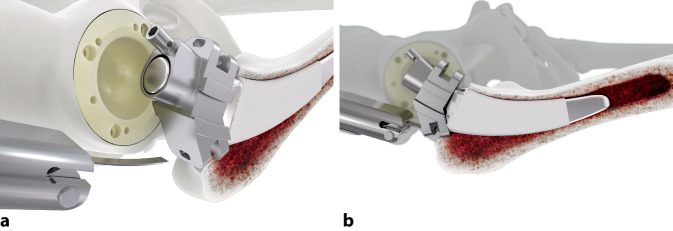

**Figure 9:**
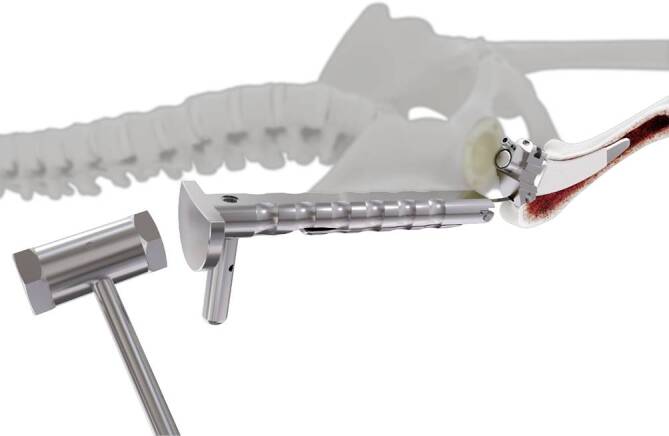

**Figure 10:**
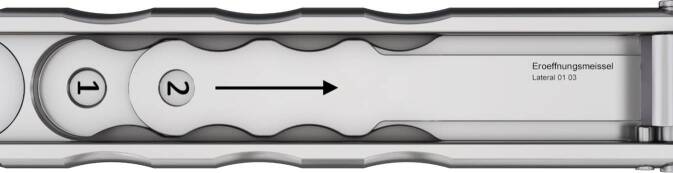

**Figure 11:**
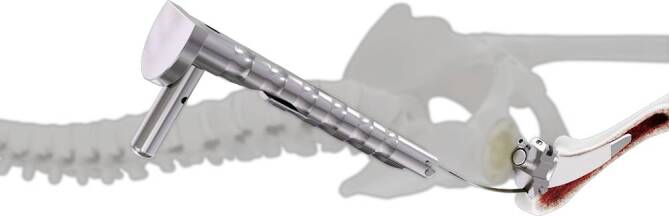

**Figure 12:**
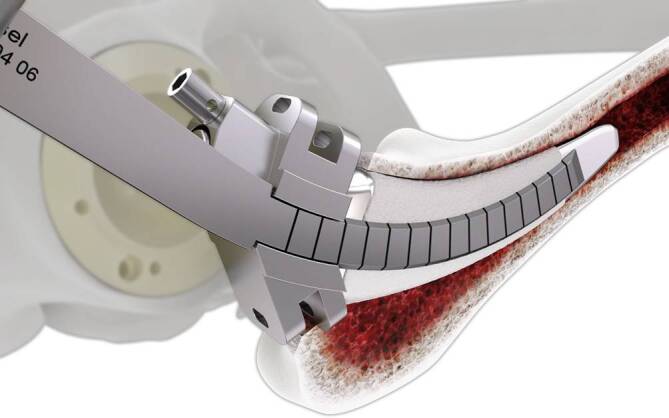

**Figure 13:**
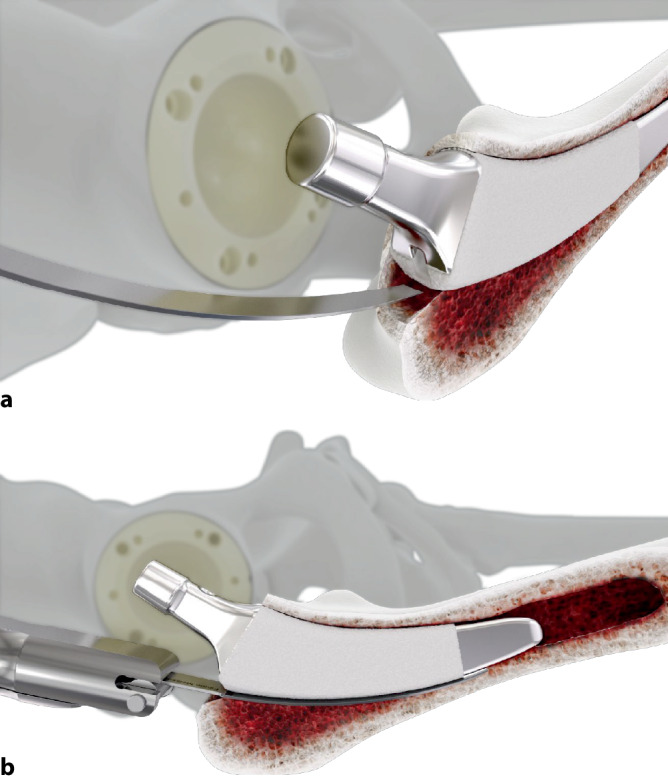

**Figure 14:**
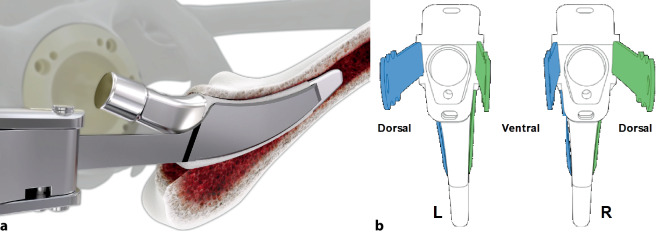

**Figure 15:**
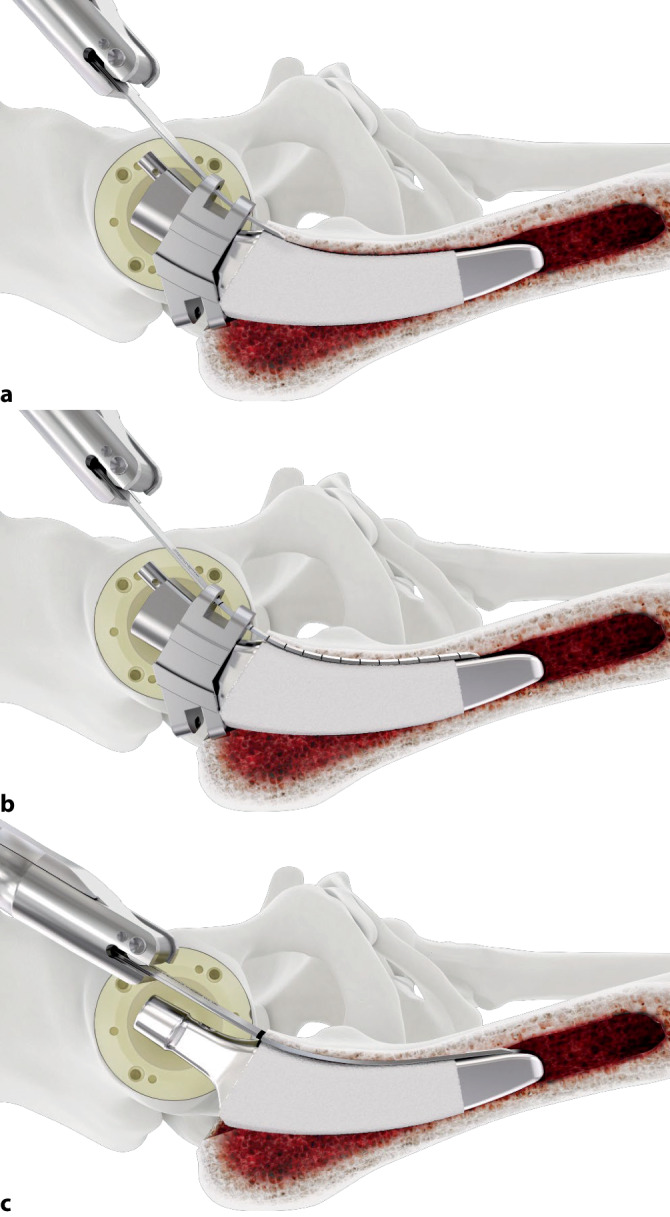

**Figure 16:**
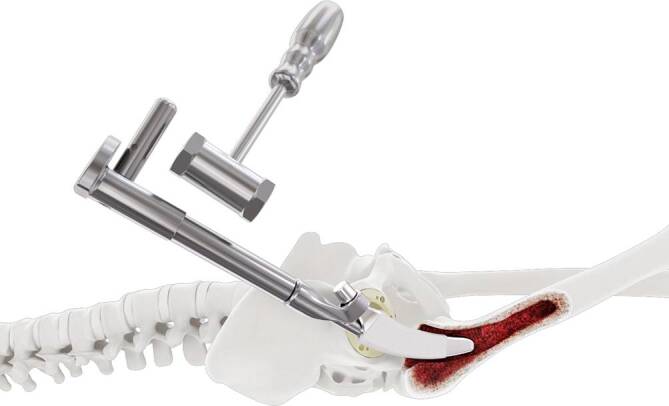

## Besonderheiten

- Die ventralen bzw. dorsalen Meißel können, abhängig davon, ob die Operation am rechten oder am linken Hüftgelenk erfolgt, jeweils sowohl als ventrale als auch als dorsale Meißel genutzt werden (s. auch Abb. 14b).

## Postoperative Behandlung

- Mobilisierung und Belastungsaufbau gemäß dem verwendeten Implantat bzw. der Verankerungstechnik

## Fehler, Gefahren, Komplikationen und ihre Behandlung

- Frustrane Ausschlagversuche nach mehrfacher systematischer Anwendung des Extraktions-Meißel-Systems: Verfahrenswechsel und ggf. Fensterung bzw. transfemoraler Zugang
- Perforation der Kortikalis, insbesondere mit dem Finalen Meißel: Verfahrenswechsel und ggf. Fensterung bzw. transfemoraler Zugang

## Ergebnisse

Das beschriebene Vorgehen hat sich in der klinischen Praxis in den 3 Autorenkliniken in insgesamt 14 Fällen bewährt. In 3 (21,4 %) Fällen musste trotz Nutzung des Extraktions-Meißel-Systems zusätzlich ein transfemoraler Zugang bzw. eine Fensterung erfolgen, um das Schaftimplantat zu entfernen. Diese Fälle traten insbesondere während der ersten Versuche im Rahmen der Lernkurve mit dem Extraktionssystem auf. Hierbei kam es beispielsweise zu einer Verwechslung der entsprechenden Meißel, sodass der Schliff nach innen, zum Implantat gewandt, ausgerichtet war. Somit konnte das Interface nicht ausreichend gelöst werden, und das Ausschlagen verlief frustran. Insbesondere mit dem medialen Meißel kam es sowohl zu einer Perforation sowie zu einer drohenden Perforation der Kortikalis. In einigen Fällen konnte eine erfolgreiche Extraktion jedoch auch ohne die Anwendung der medialen Meißel erfolgen.

Es ist für die Nutzung des Extraktions-Meißel-Systems keine zusätzliche Erweiterung des Zugangs zum Hüftgelenk nötig, und es entsteht kein zusätzlicher Weichteilschaden. Die technische Schwierigkeit ist gering, die Führung durch die Schablone erhöht die Sicherheit maßgeblich. Zum Teil ist eine deutliche Zeitersparnis möglich im Vergleich zur Nutzung konventioneller Meißel. Die Operationszeit kann sich im Vergleich zu einem transfemoralen Zugang jedoch auch zum Teil erheblich verlängern durch das aufwändige und teilweise wiederholte Freimeißeln in entsprechender Reihenfolge.

Die Indikationen, welche zur Extraktion führten, waren in der Mehrzahl periprothetische Infektionen (10 Fälle; 63,6 %), gefolgt von Malpositionen (4 Fälle; 36,4 %). In allen Fällen von periprothetischer Infektion erfolgte ein zweizeitiges Vorgehen entweder unter vorübergehender Anlage einer Girdlestone-Situation oder Spacerimplantation. Als Revisionsimplantate wurden nach Extraktion mittels des Meißelsystems in 2 (18,2 %) Fällen ein zementfreier Revisionsschaft, in 4 (36,4 %) Fällen ein zementfreier, konventioneller Primärschaft und in 3 (21,4 %) Fällen ein zementierter, konventioneller Schaft implantiert. In 4 (36,4 %) Fällen konnte erneut ein zementfreier Kurzschaft implantiert werden (Abb. 17a–c). In 1 Fall (9,1 %) einer periprothetischen Infektion wurde die Girdlestone-Situation belassen.

**Figure 17:**